# Dieterich Disease: Avascular Necrosis of the Third Metacarpal Head in an Adult

**DOI:** 10.3390/life16091440

**Published:** 2026-08-29

**Authors:** Cing-Syue Lin, Wei-Ting Wang

**Affiliations:** Department of Orthopedic Surgery, Far Eastern Memorial Hospital, New Taipei City 220, Taiwan; salantfg@gmail.com

**Keywords:** Dieterich disease, metacarpal head, avascular necrosis, bone graft

## Abstract

Background: Dieterich disease, or avascular necrosis of the metacarpal head, is a rare condition predominantly affecting adolescents, with fewer than 60 reported cases. We report a rare presentation in a 59-year-old adult with multiple systemic comorbidities. Methods: A 59-year-old man with diabetes, hypertension, and chronic kidney disease presented with progressive right third metacarpophalangeal joint pain without prior trauma. Following radiographic and magnetic resonance imaging (MRI) evaluation, the patient underwent surgical curettage of the necrotic bone followed by autologous iliac cancellous bone grafting. Histopathological examination supported the diagnosis. Results: At the long-term nearly 5-year follow-up, the patient maintained complete symptom resolution, fully restored range of motion, and durable osseous graft integration without secondary collapse or osteonecrotic recurrence. Conclusions: Although most frequently described in adolescents, Dieterich disease should be considered in adults presenting with unexplained metacarpophalangeal joint pain, especially alongside vascular-compromising comorbidities. MRI serves as a valuable adjunct for early lesion detection when radiographs are inconclusive. In this patient, joint-preserving curettage with autologous bone grafting provided durable structural support and favorable long-term functional recovery.

## 1. Introduction

Dieterich disease is a rare form of avascular necrosis affecting the metacarpal head. Unlike avascular necrosis at more commonly affected sites, such as the femoral head or the lunate in Kienböck disease, it remains underrecognized by orthopedic surgeons, hand surgeons, and radiologists alike [1]. Its rarity, combined with inconsistent terminology used across the sparse existing literature, has likely contributed to this diagnosis being frequently overlooked in routine clinical practice [2,3].

Most of what is currently known about this condition is derived from isolated case reports and small case series rather than from any prospective or registry-based study, an inherent limitation of exceedingly rare musculoskeletal conditions. Dieterich disease has traditionally been described as predominantly affecting adolescents and young adults, with more than half of reported cases occurring before age 20, though it can manifest across a broad age spectrum ranging from 6 to 82 years. Clinical suspicion should therefore be maintained when middle-aged or older adults present with unexplained metacarpophalangeal pain, particularly alongside microvascular comorbidities such as diabetes mellitus and chronic kidney disease, which could plausibly predispose to ischemic bone injury through mechanisms distinct from those in younger patients. The potential relationship between these comorbidities and the presentation or management of Dieterich disease remains insufficiently characterized in the available literature.

In this report, we describe Dieterich disease in a 59-year-old man with diabetes mellitus, hypertension, and stage 4 chronic kidney disease who underwent curettage and autologous cancellous bone grafting. The clinical value of this case lies in its uncommon comorbidity profile, the rationale for selecting a joint-preserving procedure in the presence of largely preserved articular cartilage, and the availability of nearly 5 years clinical and radiographic follow-up, including a QuickDASH assessment.

## 2. Case Presentation

A 59-year-old right-handed male fish vendor with a history of diabetes mellitus (HbA1c 8.2%), hypertension, and stage 4 chronic kidney disease (BUN 32 mg/dL, creatinine 2.42 mg/dL, eGFR [MDRD] 28 mL/min/1.73 m^2^) presented to our outpatient clinic with progressive pain and a subjective sense of joint fullness of the right third finger over 8 months. Prior to referral, he had undergone approximately 3 months of conservative management at a local medical clinic, including oral nonsteroidal anti-inflammatory drugs (NSAIDs), custom finger splinting, and temporary occupational activity modification. Despite adherence, his pain and functional limitation progressively worsened, interfering with his occupational tasks that required repetitive gripping and manual handling of fish crates. He denied any preceding traumatic event, occupational injury, corticosteroid use, or autoimmune disease; he reported a 40-pack-year smoking history and daily alcohol use for approximately 40 years, and there was no prior joint disease.

On presentation, physical examination revealed localized swelling, tenderness, and painful limitation of motion at the third metacarpophalangeal (MCP) joint, without overlying erythema or skin changes. Active range of motion was limited to 20 degrees to 50 degrees, compared with a full physiological range on the contralateral hand, with a Visual Analogue Scale (VAS) pain score of 6/10. Grip strength, assessed using a hand dynamometer, was reduced to approximately 60% of the contralateral hand. No joint instability, crepitus, or gross deformity was noted, and the remaining digits and wrist showed normal range of motion and alignment. Given the chronicity of symptoms and the patient’s comorbidities, the initial differential diagnosis included degenerative osteoarthritis, gouty or pseudogout arthropathy, occult metacarpal fracture, and inflammatory or infectious arthritis, all of which required systematic exclusion before considering avascular necrosis.

**Figure 1:**
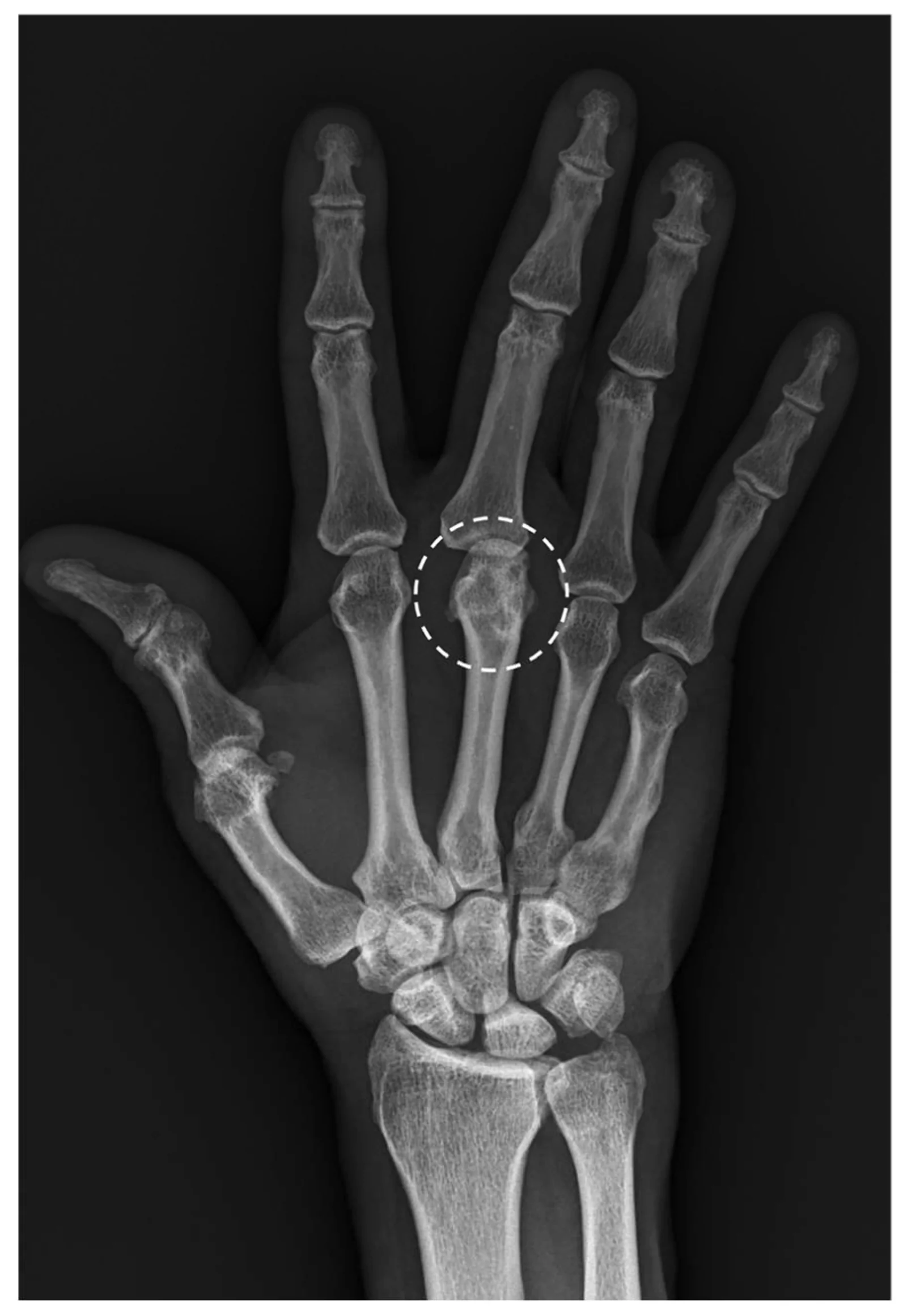
Plain radiograph of the right hand showing mixed sclerosis and subchondral lucency of the third metacarpal head (dashed circle indicates the lesion).

**Figure 2:**
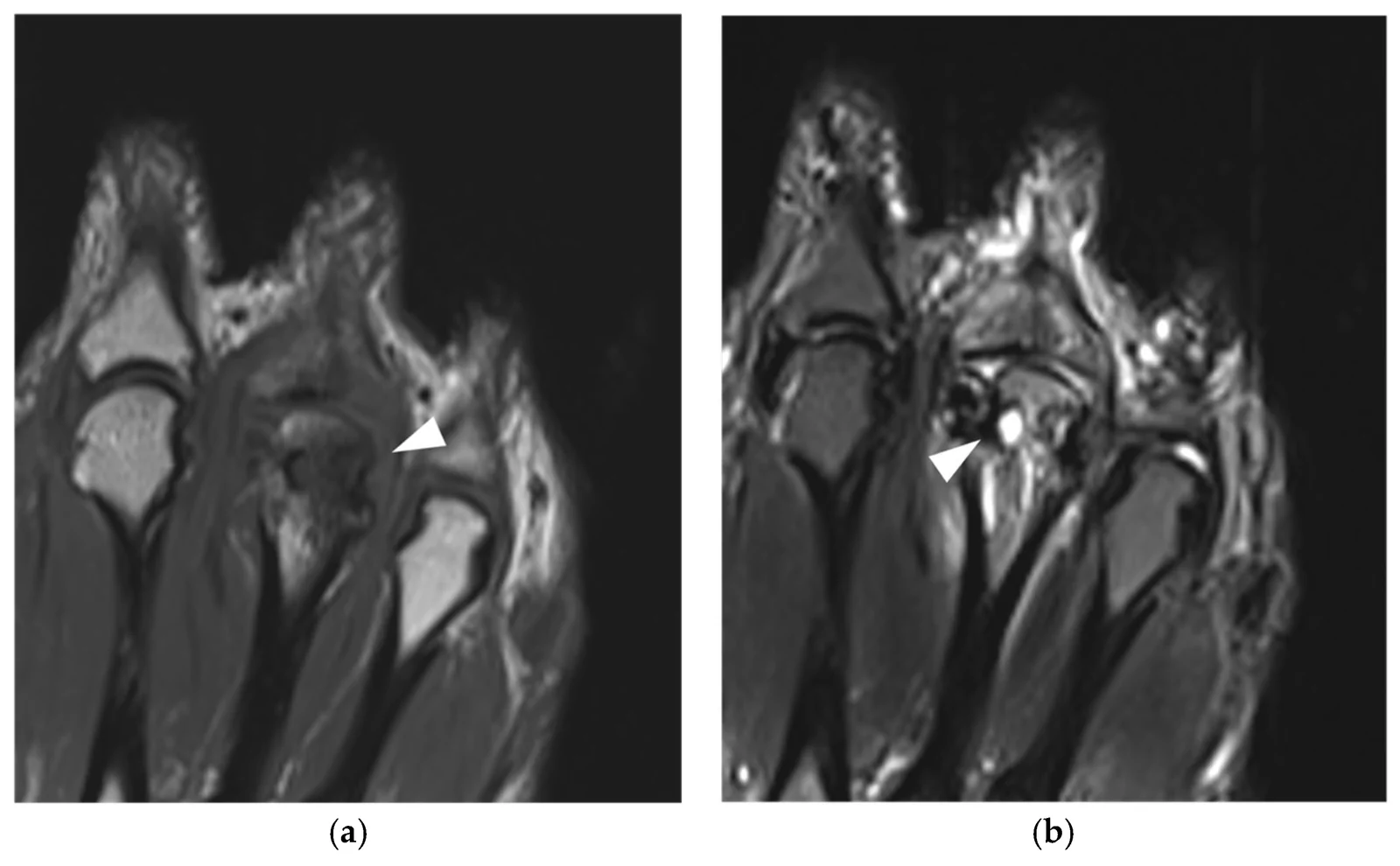
Magnetic resonance imaging (MRI) of the right hand demonstrating subchondral hypointensity on the coronal T1-weighted sequence ((**a**), arrowhead) and corresponding subchondral hyperintensity on the coronal T2-weighted fat-suppressed sequence ((**b**), arrowhead) within the third metacarpal head, consistent with avascular necrosis.

Laboratory investigations, including complete blood count, C-reactive protein, erythrocyte sedimentation rate, rheumatoid factor, and antinuclear antibody, were within normal limits, effectively excluding an infectious or autoimmune etiology and supporting a diagnosis of a primary bone ischemic process rather than an inflammatory arthropathy. Plain radiographs of the right hand demonstrated mixed subchondral sclerosis and lucency of the third metacarpal head, without gross joint space narrowing or periarticular erosive changes (Figure 1). Given the nonspecific nature of these findings, MRI was obtained and demonstrated subchondral hypointensity on T1-weighted and corresponding hyperintensity on T2-weighted sequences within the third metacarpal head, findings favoring avascular necrosis, without evidence of an underlying soft tissue mass, marrow infiltration, or neoplastic or infectious process (Figure 2a,b).

Given the failure of conservative treatment, persistent pain, occupational restriction, and radiologic evidence of subchondral osteonecrosis, surgical intervention was recommended. Continued conservative observation was deemed inappropriate due to the imminent risk of secondary subchondral collapse. Among joint-preserving surgical options, core decompression alone was deemed inadequate for an established subchondral defect with necrotic bone fragmentation, while corrective osteotomy would not evacuate the necrotic focus. Curettage with autologous cancellous bone grafting was selected to directly evacuate non-viable bone tissue and provide structural support to the overlying cartilage. The patient elected to proceed with surgery given his desire for a more definitive and expedited functional recovery.

Under general anesthesia with the patient positioned supine, autologous cancellous bone graft was first harvested from the right anterior superior iliac spine using a standard iliac crest harvesting technique. The right third MCP joint was then exposed through a longitudinal dorsal approach, with careful preservation of the extensor mechanism. Intraoperatively, the overlying articular cartilage was found to be largely intact, with only focal fissuring along the dorsal aspect, supporting the feasibility of a joint preservation strategy rather than a more extensive reconstructive procedure. Following arthrotomy, thorough curettage of the necrotic subchondral bone was performed until viable, bleeding cancellous bone was encountered at the defect margins. The resulting cavity was densely packed with the harvested iliac graft to support the overlying cartilage, followed by layered capsular repair and standard wound closure.

**Figure 3:**
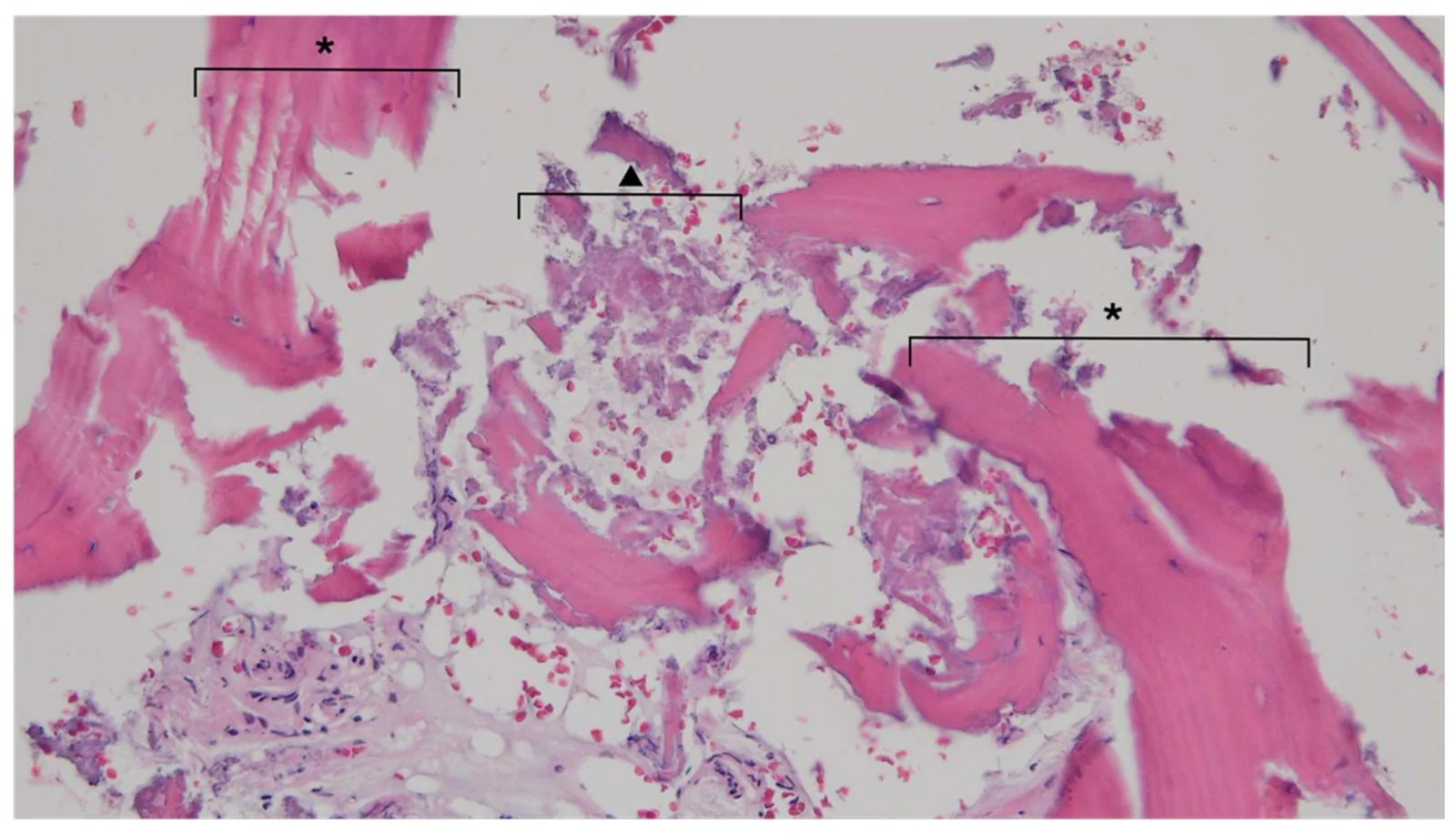
Histopathological examination of the curetted specimen showing fragmented woven bone with focal ragged/devitalized bone (asterisks) and fibroplasia within the marrow spaces (arrowhead). No malignancy was identified. In conjunction with the clinical and imaging findings, these features were compatible with avascular necrosis (hematoxylin and eosin stain, original magnification ×200).

Histopathological examination of the curetted specimen revealed fragmented woven bone with focally ragged and devitalized bone, along with focal fibroplasia within the marrow spaces, without evidence of malignancy, compatible with avascular necrosis (Figure 3), supporting the clinical and radiographic impression. Postoperatively, the hand was immobilized in a short-arm splint for 3 weeks to protect the graft site, after which the patient transitioned to a structured, supervised hand therapy program emphasizing progressive range-of-motion exercises, edema control, and gradual return to functional grip strengthening. The patient tolerated rehabilitation well, without wound complications, graft extrusion, or signs of infection.

**Figure 4:**
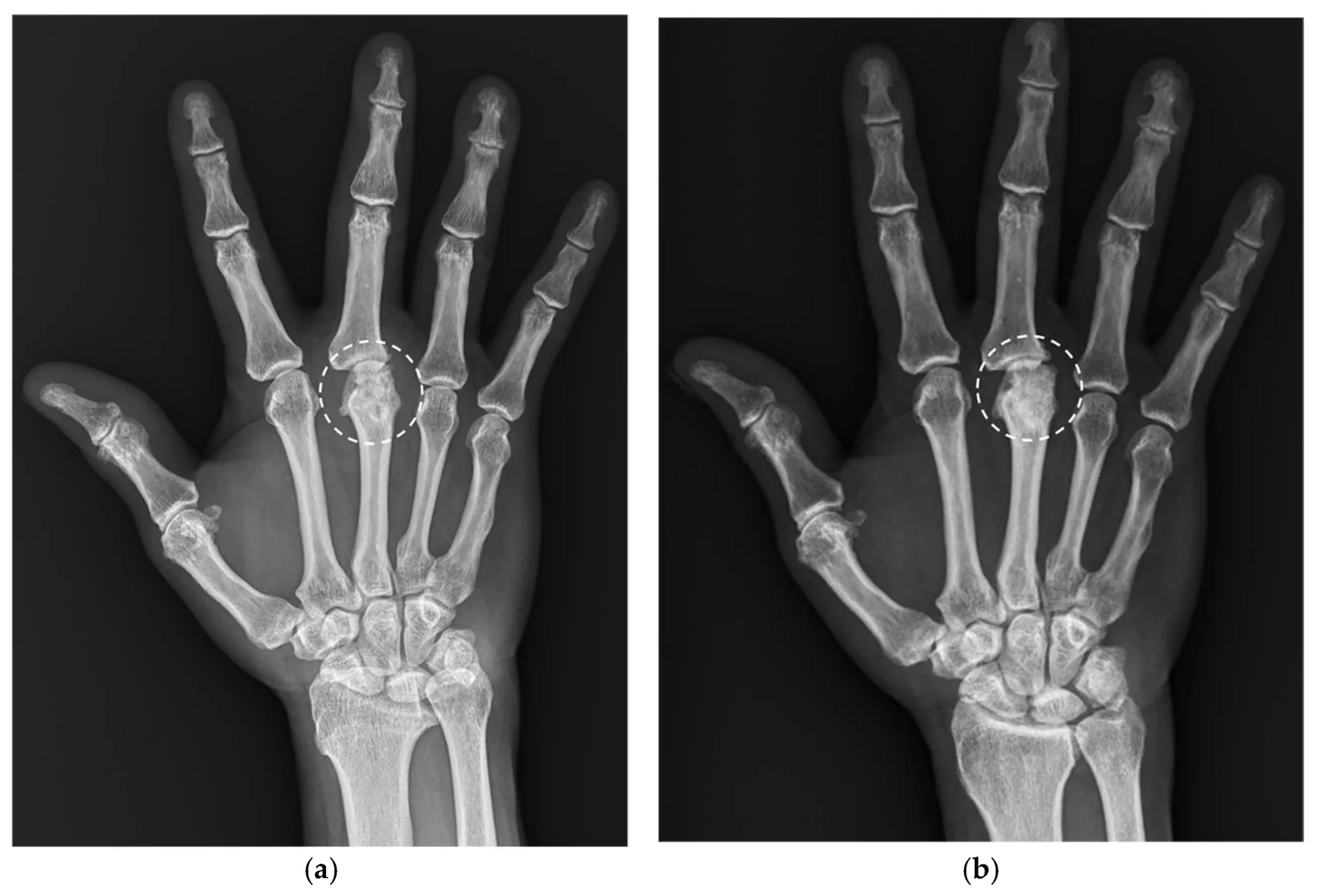
(**a**) Follow-up radiograph at postoperative 3 months showing progressive bone regeneration without collapse (dashed circle indicates the lesion). (**b**) Long-term follow-up radiograph at nearly 5 years (58 months) postoperatively showing complete bony integration of the autologous graft and progressive osseous regeneration of the third metacarpal head (dashed circle), without secondary collapse, articular degradation, or osteonecrotic recurrence.

At the 3-month postoperative follow-up, the patient reported significant symptomatic improvement, with a VAS pain score reduced to 1/10, and active range of motion improved to 5 degrees to 85 degrees, with grip strength also subjectively improved and approaching that of the contralateral hand. He had successfully returned to his prior occupational duties without functional restriction. Follow-up radiographs at 3 months postoperatively demonstrated progressive osseous regeneration of the metacarpal head, with maintained joint congruity and no evidence of subchondral collapse or graft resorption (Figure 4a), consistent with successful bony incorporation of the graft.

At the nearly 5-year postoperative follow-up (around 58 months postoperatively), the patient maintained good long-term clinical and functional recovery. He reported VAS pain score of 0/10, a pain-free range of motion of the third MCP joint, equivalent to the contralateral side, and grip strength measured 33 kg on the operated (right) hand versus 36 kg on the contralateral (left) hand, approximately 92% of the contralateral side. His QuickDASH score markedly improved from a baseline of 45.5/100 to 5.6/100. Radiographs obtained at nearly 5 years also demonstrated durable bony integration of the graft, with no secondary osteonecrosis recurrence or subchondral collapse (Figure 4b).

## 3. Discussion

Dieterich disease, first described by Mauclaire in 1927 and further characterized by Dieterich in 1932 [1,4], remains an exceedingly rare cause of metacarpophalangeal joint pain, with fewer than 60 cases reported worldwide to date [1]. Epidemiologically, the condition demonstrates a male-to-female predominance of approximately 3:2, most commonly affects the long (middle) finger metacarpal head (46%), followed by the index and ring fingers (19% each), with the thumb least frequently involved (5%) [5]. Although classically described in adolescents and young adults, with more than half of reported cases occurring before the age of 20, the disease has been documented from as young as 6 years to as old as 82 years, indicating that clinicians should maintain suspicion regardless of patient age [6,7] (Table 1). While prior literature has largely focused on young, healthy individuals without systemic illness, our case adds a distinct adult perspective, detailed further below.

When compared with previously reported adult cases (Table 1), the existing literature reflects a wide therapeutic spectrum tailored to patient demand and articular status, ranging from conservative management and joint denervation to curettage-based reconstruction and salvage arthroplasty [6,8,9,10,11]. Our case adds to this literature across three dimensions.

**Table 1:** Summary of selected published adult cases of Dieterich disease compared with the present case.

Study	Age (Years)/Sex	Comorbidities	Finger Involved	Treatment	Outcome
Bailey et al. (2021) [8]	23/M	None reported	Ring (fourth)	MCP joint denervation (neurectomy)	80% pain relief, QuickDASH improved from 45 to 10 at 2.5-year follow-up
Kim et al. (2018) [11]	29/F	None reported	Long (third)	Pyrolytic carbon hemiarthroplasty (for advanced cartilage erosion)	Satisfactory pain-free full ROM at 1-year follow-up
Hu et al. (2008) [9]	51/M	None reported	Middle (third)	Curettage + multiple drilling (without bone graft)	Pain-free with full ROM (0–80°) at 1-year follow-up
Maryada et al. (2018) [10]	57/M	None reported	Index	Curettage + cancellous graft (distal radius) after failed conservative trial	Symptom-free at 1-year follow-up
Martínez Núñez et al. (2021) [6]	82/M	Not specified	Third (middle)	Conservative (NSAIDs)	Significant clinical improvement, no functional limitation
Present case (2026)	59/M	Diabetes mellitus, hypertension, stage 4 CKD	Middle (third)	Curettage + autologous iliac crest bone graft	Pain-free function, QuickDASH 5.6/100, and radiographic graft integration without secondary collapse at 58-month follow-up

M = male; F = female; ROM = range of motion; CKD = chronic kidney disease; MCP = metacarpophalangeal; QuickDASH = Quick Disabilities of the Arm, Shoulder, and Hand.

The first concerns comorbidity profile: systemic metabolic disease was either not reported or not emphasized in the selected adult cases summarized in Table 1. Our patient represents an uncommon clinical context in which Dieterich disease occurs alongside concurrent stage 4 chronic kidney disease and diabetes mellitus; as this remains a single observation, a causal relationship between these comorbidities and metacarpal head osteonecrosis cannot be inferred.

A second dimension lies in how the extent of cartilage damage guides surgical selection. Kim et al. [11] proceeded to pyrolytic carbon hemiarthroplasty after intraoperative findings of diffuse cartilage erosion left little viable articular surface. Bailey et al. [8], treating a 23-year-old professional acrobat who wished to preserve joint flexibility for his occupation, opted for MCP joint denervation rather than an intra-articular procedure. Hu et al. [9] encountered focal, well-demarcated bare bone at the subchondral surface and addressed it with curettage and multiple drilling without bone grafting. In our patient, as in Maryada et al. [10], the overlying cartilage remained largely intact, supporting curettage with autologous cancellous bone grafting as the joint-preserving option of choice.

The third dimension concerns follow-up duration; reported postoperative follow-up among the selected surgically treated adult cases ranged from 1 to 2.5 years (Hu et al. [9]: 1 year; Kim et al. [11]: 1 year; Maryada et al. [10]: 1 year; Bailey et al. [8]: 2.5 years). Our study provides a longitudinal follow-up spanning nearly 5 years (58 months postoperatively), documenting maintained radiographic graft incorporation and good functional recovery, including a QuickDASH score of 5.6/100, in this patient despite the presence of systemic comorbidities.

The vascular anatomy of the metacarpal head offers a plausible pathogenetic explanation: in roughly one-third of cadaveric specimens, no single dominant vessel supplies the distal epiphysis, leaving the metacarpal head reliant instead on a network of fine pericapsular arterioles for its blood supply [4,5]. While avascular necrosis in the hand and wrist routinely affects the lunate or scaphoid, and occasionally other carpal bones [2], its manifestation in the metacarpal head remains exceedingly rare. Beyond a single occult fracture, chronic repetitive microtrauma from sustained gripping and load-bearing activities, such as those involved in fish handling, may gradually injure the pericapsular vessels, even without any single memorable traumatic event. Although this mechanism cannot be established in the present case, the patient’s occupational history of repetitive manual handling may represent a possible mechanical contributor within a multifactorial disease process, involving anatomic, genetic, and environmental factors [4,5].

Clinically, Dieterich disease presents with a broad and often nonspecific spectrum of severity: pain is the most common presenting symptom (87%), followed by edema (49%) and restricted range of motion (49%), while crepitus is comparatively uncommon (8%) [4]. The disease may also remain entirely asymptomatic and be discovered incidentally, as illustrated by an 82-year-old patient whose metacarpal head showed advanced radiographic changes despite minimal functional limitation [6]. A history of antecedent trauma, often trivial or unrecognized, has been reported in some cases, and clinical examination frequently reveals only subtle dorsal swelling or tenderness without gross deformity or instability [3,5]. This variability reinforces the importance of maintaining a high index of clinical suspicion in patients with unexplained, localized metacarpophalangeal pain unaccompanied by systemic inflammatory findings [4,12].

Establishing the diagnosis of Dieterich disease relies heavily on images, particularly when early plain radiographs are subtle or equivocal [4,7]. In our patient, MRI showed the classic pattern of subchondral T1 hypointensity and T2 hyperintensity, favoring early avascular necrosis when radiographs showed only non-specific changes, and additionally demonstrated preservation of the overlying articular cartilage [13]. This finding was clinically important, as cartilage integrity directly informed the decision to proceed with a joint-preserving reconstructive approach rather than a joint-sacrificing salvage procedure [1,14].

No universally accepted treatment algorithm exists for Dieterich disease given the rarity of reported cases. Nonsurgical management, comprising rest, splint immobilization, and nonsteroidal anti-inflammatory drugs for 3 to 6 months, is generally recommended as first-line therapy, particularly in pediatric and adolescent patients who retain greater intrinsic remodeling potential [4,12]. In a review of 31 pooled cases, approximately 39% of patients were successfully managed nonoperatively, while the remaining 61% ultimately required surgical intervention after failure of conservative measures [4], underscoring the importance of close radiographic follow-up during any conservative trial.

For adults with persistent symptoms or progressive collapse, surgical options include core decompression, curettage with bone grafting, flexion osteotomy, osteochondral autograft transplantation, and arthroplasty [1,4]. Selection depends on disease stage, cartilage integrity, and the extent of subchondral necrosis. Isolated core decompression can relieve ischemia in early, pre-collapse stages, but it is rarely used alone once structural changes such as subchondral sclerosis, lucency, and fragmented trabeculae have developed, since it cannot evacuate necrotic debris or restore structural support [1]. Curettage combined with autologous cancellous bone grafting, by contrast, directly removes devitalized tissue and restores subchondral mechanical integrity; when the overlying cartilage remains largely preserved, this approach may provide pain relief and preserve joint structure without sacrificing the joint [1,10]. This rationale was supported in our patient by intact dorsal cartilage at surgery and by the favorable structural and functional outcome at nearly 5 years. Other options, including flexion osteotomy, vascularized bone transfer, and, for advanced joint destruction, arthroplasty or arthrodesis, remain viable alternatives depending on cartilage status and disease severity [1,2,4]. Diabetes and chronic kidney disease are both plausible, though unproven, risk factors for microvascular vulnerability and impaired bone remodeling [15], potentially compounding local perfusion deficits from repetitive manual loading. Perioperative glycemic and renal optimization was therefore prioritized; his clinical course nonetheless showed no delayed incorporation or nonunion.

A structured hand therapy program after protective splinting allowed progressive restoration of range of motion and grip strength without compromising graft stability, consistent with rehabilitation protocols described in other successfully treated cases [5,10]. A practical implication of this case is the value of validated outcome instruments such as the QuickDASH: while subjective symptom relief is reassuring, a quantitative score provides a degree of objectivity that is frequently absent from case reports of this kind. Treatment selection should therefore be individualized based on patient age, symptom duration, cartilage integrity on imaging, and the presence of comorbidities that may influence bone healing potential, with a stepwise approach, beginning with conservative management where feasible and reserving joint-preserving surgery for persistent symptoms or radiographic progression, remaining the most pragmatic strategy given the current absence of high-level comparative evidence.

While the single-case design inherently limits generalizability, curettage combined with autologous cancellous bone grafting achieved durable osseous integration and near-complete functional recovery despite microvascular and bone-healing compromises from diabetes and chronic kidney disease. This outcome reinforces that Dieterich disease is not exclusively a pediatric or adolescent condition [7] and supports the feasibility of joint-preserving reconstruction in adults with systemic comorbidities.

## 4. Conclusions

In conclusion, this report reinforces that Dieterich disease, while most frequently recognized in pediatric and adolescent populations, can also manifest in middle-aged and older adults, in whom microvascular and metabolic comorbidities such as diabetes mellitus and chronic kidney disease were notable clinical features, although their contribution to the development of Dieterich disease remains uncertain. This highlights the value of MRI in this case for identifying the lesion when plain radiographic findings were equivocal and for assessing articular cartilage integrity to guide treatment selection, and further underscores Dieterich disease as a differential diagnosis for unexplained metacarpophalangeal pain across all age groups. Our findings also support joint-preserving surgery, curettage with autologous bone grafting, as a feasible and durable treatment option when articular cartilage integrity is preserved, with sustained structural stability and functional recovery demonstrated over nearly 5 years in this case. Continued case reporting and, where feasible, pooled multicenter analyses remain essential to better define this condition’s natural history, treatment thresholds, and long-term outcomes across diverse patient populations.

## Data Availability

The data presented in this case report, including the patient’s clinical and follow-up imaging data, are not publicly available due to patient privacy considerations but are available from the corresponding author upon reasonable request.

## Abbreviations

The following abbreviations are used in this manuscript:

MCP joint	Metacarpophalangeal joint
MRI	Magnetic resonance imaging
ASIS	Anterior superior iliac spine
IRB	Institutional Review Board
QuickDASH	Quick Disabilities of the Arm, Shoulder, and Hand
